# Prevalence of Obesity and Its Relationship With Hypertension Among School-Going Adolescents Aged 12–16 Years

**DOI:** 10.7759/cureus.42999

**Published:** 2023-08-05

**Authors:** Suba Rajinikanth B, Sujatha U, Sankalp Yadav

**Affiliations:** 1 Paediatrics, Faculty of Medicine - Sri Lalithambigai Medical College and Hospital, Dr. MGR Educational and Research Institute, Chennai, IND; 2 Medicine, Shri Madan Lal Khurana Chest Clinic, New Delhi, IND

**Keywords:** body mass index: bmi, adolescents, childhood obesity, hypertension, obesity

## Abstract

Background: Childhood obesity is a significant health issue that has grown in prominence, particularly in developed countries. Primary hypertension has become the dominant cause, leading to an increased incidence of arterial hypertension. This study examined the associations between sex and obesity with hypertension (HTN). Understanding these associations can provide insights into the risk factors and potential preventive strategies for HTN.

Materials and methods: This cross-sectional observational study was conducted at private schools in Chennai, Tamil Nadu, India, for one year. Data analysis was performed on a cohort of participants who underwent health assessments, including blood pressure measurements, self-reported dietary habits, and socio-economic status information. Statistical analyses assessed the associations between sex, dietary habits, socio-economic status, and HTN.

Results: There were 255 (51.0%) males and 245 (49%) females. Among the female participants, only two individuals (0.8%) had HTN, while among males, 11 individuals (4.3%) had HTN, indicating a significant association between sex and HTN (P=0.014). In terms of dietary habits, the prevalence of HTN was similar among non-vegetarians (2.5%) and vegetarians (3.1%), and the association was not statistically significant (P=0.777). Among the obese individuals in the study population, eight individuals (8.8%) had HTN, while the remaining 83 individuals (91.2%) did not have HTN, with a P-value of <0.0001, which indicates a significant association between HTN and obesity.

Conclusion: This study revealed a significant association between sex and HTN, with males exhibiting a higher prevalence of HTN than females. Furthermore, the study showed a significant association between obesity and hypertension, indicating that obese individuals were more likely to have HTN.

## Introduction

In developed countries, childhood obesity has been a significant health issue. According to statistics from 2017 to 2020, the prevalence of obesity among children and adolescents aged 2-19 years was 19.7%, affecting approximately 14.7 million individuals [1]. The prevalence of obesity varied across different age groups, with rates of 12.7% among 2- to 5-year-olds, 20.7% among 6- to 11-year-olds, and 22.2% among 12- to 19-year-olds [1]. The World Health Organization (WHO) reports that the prevalence of overweight and obesity in children and adolescents aged 5-19 has dramatically increased. In 1975, the combined rate was only 4%, but by 2016, it had risen to just over 18%. This increase occurred similarly among both boys and girls, with 18% of girls and 19% of boys classified as overweight in 2016 [2]. In contrast, the prevalence of obesity among children and adolescents aged 5-19 was less than 1% in 1975 but had risen to affect over 124 million individuals (6% of girls and 8% of boys) in 2016.

In recent years, there has been a significant shift in the primary causes of hypertension (HTN, high blood pressure) in children over the age of 6 [3]. Previously, secondary causes such as kidney disorders were more prevalent, but now primary hypertension has become the dominant cause, particularly among older schoolchildren. This change is attributed to the global epidemic of obesity that has been observed over the past decade [4]. The rise in obesity rates has led to an increased incidence of arterial hypertension, making it one of the most common health issues in children's development. Studies have found that indicators of obesity, such as body mass index (BMI) and other obesity indexes, can help identify children with elevated blood pressure levels [5]. These measurements are useful tools for assessing the risk of hypertension and associated health problems in children. It is important to address the link between obesity and hypertension in children, as early identification and intervention can play a crucial role in preventing long-term health complications.

The study aimed to investigate the prevalence of obesity and hypertension in the target population and explore the potential relationship between obesity and hypertension. For these, measurements such as waist circumference and BMI were assessed.

## Materials and methods

This cross-sectional observational study was conducted after ethical committee approval (Dr.MGR-ERI/SLMCH/2022/009) on 500 adolescent school-going girls and boys at private schools in Chennai, Tamil Nadu, for one year. The sample size was determined based on a pooled data estimate of a 19.7% combined prevalence of childhood overweight and obesity after 2010.

Inclusion criteria

Adolescent school-going girls and boys aged 12 to 16 years and prior informed consent obtained from the participants or their guardians were included.

Exclusion criteria

Adolescents with known genetic or syndromic abnormalities, adolescents with chronic systemic medical illnesses related to cardiac, renal, neurologic, etc., and adolescents or their guardians who refused consent were excluded.

A standardized proforma collected participants' information, including demographic details and anthropometric measures. Vitals and blood pressure examinations were conducted and meticulously recorded as part of the data collection process. Height measurements were taken using a standardized stadiometer placed on a level surface. Participants removed shoes, socks, and hair ornaments and stood with their feet slightly apart on the baseboard. The back of the head, shoulder blades, buttocks, calves, and heels touched the vertical board. Trunk balance, straight legs, and flat feet were ensured. The head was aligned parallel to the baseboard, and the headboard was gently pressed on top, compressing the hair. The height was recorded in centimeters, rounded to the nearest 0.1 cm.

The weight measurements were taken using standardized electronic weighing scales. Participants were instructed to stand in the middle of the scale with their feet slightly apart, ensuring the display showed 0.0. They were then asked to remain still until their weight appeared on the display. Weight measurements were recorded to the nearest 0.1 kg. The recorded height and weight data were plotted on the Indian Academy of Pediatrics (IAP)-modified WHO growth charts, and the corresponding percentiles were noted.

The waist circumference (WC) was measured using stretch-resistant tape. The measurements were taken at the end of several consecutive natural breaths. The tape was positioned parallel to the floor, at a midpoint between the top of the iliac crest and the lower margin of the last palpable rib in the mid-axillary line. Hip circumference was also measured using stretch-resistant tape. The tape was wrapped around the participant at the largest circumference of the buttocks. It should be snug but not constricting. The measurement was taken parallel to the floor. During both measurements, the participant stood upright with arms relaxed at the sides, feet evenly spread apart, and body weight evenly distributed. WC was compared to age- and gender-specific reference curves based on the Indian population. A WC above the 90th centile indicated an increased risk for metabolic and cardiovascular morbidities.

The waist-hip ratio was calculated by dividing waist circumference (cm) by hip circumference (cm). A waist-hip ratio of >0.5 predicts cardiovascular disease in adults. The same is true for adolescents as well. With the correct cuff size, blood pressure was measured in the right arm for consistency of practice. Prior permission was obtained from school authorities and parents through informed consent.

Statistical analysis

All the data from the research subjects were entered into a Microsoft Excel spreadsheet (Microsoft® Corp., Redmond, WA). Statistical Package for the Social Sciences (SPSS, IBM Corp., Armonk, NY) was used to analyze the data. Categorical variables were represented as percentages and numbers. The chi-square test was used to compare categorical variables across study groups. A P-value of 0.05 was considered statistically significant.

## Results

There were 255 (51.0%) males and 245 (49%) females. Most participants, 87.2% (436), identified themselves as non-vegetarian (NV), whereas 12.5% (64) reported being vegetarian (V). Regarding dietary habits, a significant portion of participants, 90.2% (451), reported consuming junk food, while 9.8% (49) did not consume junk food. Regarding body weight, 18.2% (91) were classified as obese, while the majority, 81.8% (409), were not obese. Similarly, 22.6% (113) were categorized as overweight, and 77.4% (387) were not. The prevalence of hypertension among participants was low, with only 2.6% (13) diagnosed with the condition, while the majority, 97.4% (487), did not have hypertension. Additionally, 5.4% (27) were found to have pre-hypertension, whereas 94.6% (473) did not exhibit pre-hypertensive conditions. Regarding socioeconomic status, 53.6% (268) belonged to the lower-middle class, while 46.4% (232) belonged to the upper-middle class (Table 1).

**Table 1:** Demographic data of the study V: vegetarian, NV: non-vegetarian, HTN: hypertension, pre-HT: pre-hypertension

Study variables		Frequency	Percentage
Sex	Male	255	51.0%
	Female	245	49.0%
V/NV	NV	436	87.2%
	V	64	12.5%
Junk food	No	49	9.8%
	Yes	451	90.2%
Obesity	No	409	81.8%
	Yes	91	18.2%
Overweight	No	387	77.4%
	Yes	113	22.6%
HTN	No	487	97.4%
	Yes	13	2.6%
Pre-HT	No	473	94.6%
	Yes	27	5.4%
Socio economic status	Lower middle	268	53.6%
	Upper middle	232	46.4%

Two hundred and forty-three (99.2%) females did not have HTN, while two females (0.8%) had HTN. Two hundred and forty-four (95.7%) males did not have HTN, while 11 (4.3%) males had HTN. The statistical analysis indicated a significant association between sex and HTN (P-value = 0.014). When considering dietary habits, 425 (97.5%) non-vegetarians (NV) did not have HTN, while 11 (2.5%) had HTN. Sixty-two (96.9%) vegetarians (V) did not have HTN, while two (3.1%) had HTN. This association was insignificant (P-value = 0.777). Analyzing socioeconomic status, 261 (97.4%) of the lower-middle group did not have HTN, while 7 (2.6%) had HTN. 226 (97.4%) in the upper-middle group did not have HTN, while 6 (2.6%) had HTN. This association is insignificant (P-value = 0.986). Considering junk food consumption, among participants who did not consume junk food, 46 individuals (93.9%) did not have HTN, while three individuals (6.1%) had HTN.

Among participants who consumed junk food, 441 individuals (97.8%) did not have HTN, while ten individuals (2.2%) had HTN. The statistical analysis found no significant association between junk food consumption and HTN (P-value = 0.103). In terms of overweight status, among participants who were not overweight, 377 individuals (97.4%) did not have HTN, while ten individuals (2.6%) had HTN. Among participants who were overweight, 110 individuals (97.3%) did not have HTN, while three individuals (2.7%) had HTN. The statistical analysis found no significant association between overweight status and HTN (P-value = 0.937). Lastly, among the non-obese, 404 individuals (98.8%) did not have HTN, while five individuals (1.2%) had HTN. Among the participants with obesity, 83 individuals (91.2%) did not have HTN, while eight individuals (8.8%) had HTN. The statistical analysis revealed a significant association between obesity and HTN (P < 0.0001) (Table 2).

**Table 2:** Comparison of parameters of hypertension F: female, M: male, NV: non-vegetarians, V: vegetarians

Study variables		HTN				P-value
		No		Yes		
		Frequency	Percentage	Frequency	Percentage	
Sex	F	243	99.2%	2	0.8%	0.014
	M	244	95.7%	11	4.3%	
V/NV	NV	425	97.5%	11	2.5%	0.777
	V	62	96.9%	2	3.1%	
Socio-economic status	Lower middle	261	97.4%	7	2.6%	0.986
	Upper middle	226	97.4%	6	2.6%	
Junk food	No	46	93.9%	3	6.1%	0.103
	Yes	441	97.8%	10	2.2%	
Overweight	No	377	97.4%	10	2.6%	0.937
	Yes	110	97.3%	3	2.7%	
Obesity	No	404	98.8%	5	1.2%	<0.0001
	Yes	83	91.2%	8	8.8%	

**Figure 1:**
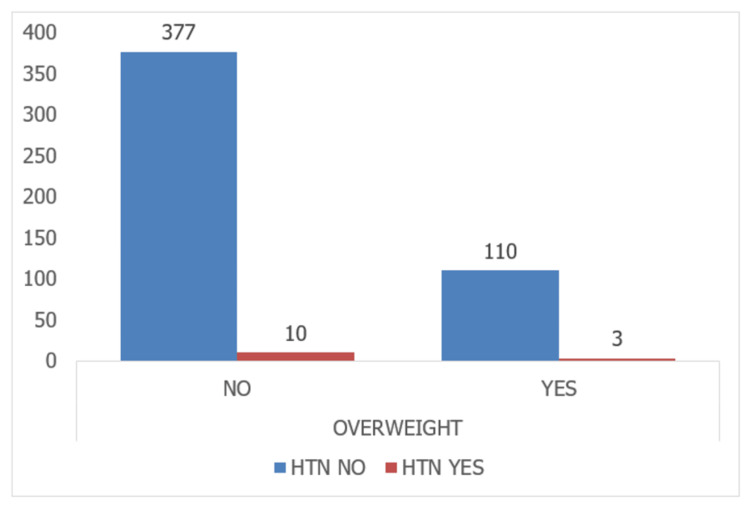
Comparison of overweight with hypertension HTN: hypertension

**Figure 2:**
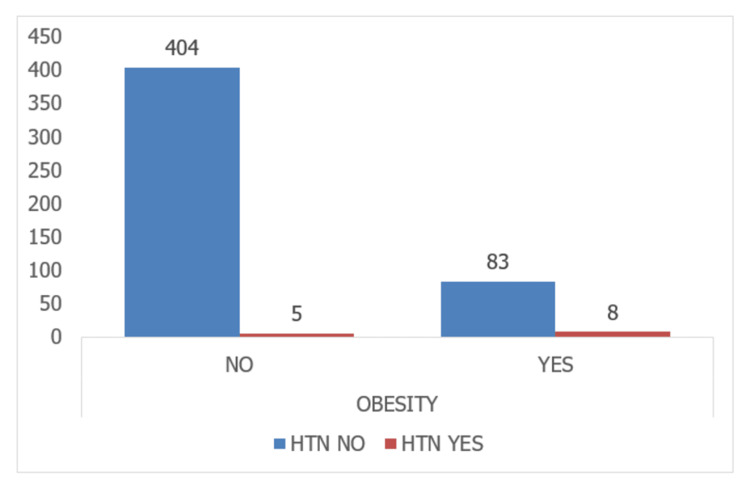
Comparison of obesity with hypertension HTN: hypertension

The prevalence of hypertension was higher in overweight and obese children than in normal-weight children in urban and rural populations, as indicated by the present study. This association has also been reported in other studies. Similar trends have been observed in studies conducted in India. The comparison of overweight with HTN and obesity with HTN is depicted in Figures 1-2, respectively.

## Discussion

Childhood obesity is a grave health issue that impacts the well-being of children and adolescents. This concern is particularly distressing as it sets children on a trajectory toward health conditions previously associated mainly with adults, such as diabetes, hypertension, and hyperlipidemia. Furthermore, childhood obesity can contribute to negative consequences like low self-esteem and depression. First, when considering sex, the current study found a significant association between sex and HTN. A higher percentage of males (4.3%) had HTN than females (0.8%). This finding suggests that males may be more susceptible to developing HTN than females in the studied population. Several studies have shown that men younger than 65 consistently have higher hypertension levels than women of the same age group [6].

The prevalence of HTN was similar among non-vegetarians (2.5%) and vegetarians (3.1%). While the association was not statistically significant, it is worth noting that the percentage of individuals with HTN was slightly higher among vegetarians. On the contrary, vegetarian dietary patterns have been associated with reductions in risk for several chronic diseases, such as hypertension and high blood pressure [7,8].

The current study found that socioeconomic status was not significantly associated with HTN. Lower-middle and upper-middle socioeconomic status groups had similar rates of HTN (2.6%). However, evidence suggests that individuals with lower socioeconomic status are more likely to engage in unhealthy behaviors, which can contribute to the development of hypertension [9,10]. In the present study, the prevalence of hypertension (HTN) was found to be slightly higher among individuals who consumed junk food (2.2%) compared to those who did not (6.1%), although this difference was statistically insignificant. These findings align with a study conducted by Zhang et al. in Southwest China, which demonstrated that higher adherence to the vegetable-grain pattern and lower adherence to the junk food pattern significantly reduced the incidence of hypertension among the population [11].

The association between obesity and hypertension is well-established, as evidenced by various studies conducted on children and adults across different genders [12,13]. In the current study, a significant relationship was observed between obesity and hypertension. Among individuals with obesity, a higher percentage (8.8%) had hypertension than those without obesity (1.2%). These findings are consistent with previous research. For example, the Framingham Offspring Study reported that a significant proportion of new cases of essential hypertension in both men and women were attributable to excess body fat (78% in men and 65% in women) [14]. Moreover, even a modest weight gain of 5% was associated with a 20-30% increase in the incidence of hypertension. In a separate Nurses' Health Study involving a large cohort of 82,882 adult women followed over 14 years, body mass index (BMI) emerged as the strongest risk factor for developing hypertension. Obese women had nearly five times the incidence of hypertension compared to those with a BMI below 23.0 kg/m2 [15].

These findings underscore the importance of addressing obesity as a significant risk factor for hypertension. The association between excess body fat and hypertension highlights the need for effective strategies for weight management, healthy lifestyle modifications, and public health interventions to combat the rising prevalence of obesity-related hypertension. As per the WHO data on childhood malnutrition, obesity is increasing in developing countries and has started plateauing in developed countries [16]. Further, in the WHO statement on regional and country-wise data on obesity and overweight, India’s major nutritional problem is mentioned in the underweight category [17]. In the general population, underweight may be the major problem, but the obesity and overweight rate is soaring and, if not given enough attention, may become a major health issue with other comorbid conditions much earlier than expected. As per the Government of India's National Family Health Survey 5 (NFHS 5), 36% of children under the age of five are stunted; 19% are wasted; 32% are underweight; and 3% are overweight [18]. India had the highest prevalence of moderate and severe underweight throughout these four decades (24.4% of girls and 39.3% of boys were moderately or severely underweight in 1975, and 22.7% and 30.7% in 2016). In 2016, 97 million of the world’s moderately or severely underweight children and adolescents lived in India [19]. This research demonstrates the importance of immediate interventions in managing the accelerated obesity epidemic in urban India, which lacks investment in government policy in India and in the global scenario as well. As most government policies and interventions are focused on tackling undernutrition, obesity and overweight prevalence in urban areas are fast catching up with undernutrition prevalence rates, and morbidity, especially hypertension at an earlier age, is highlighted to emphasize the importance of immediate action to control the obesity pandemic in India.

The study showed a strong correlation between central obesity and hypertension. Though the abnormal blood pressure was measured by different people and finally confirmed, it could not be measured on a different day in a different setting. The other reasons for hypertension need to be ruled out, which was not possible as there was no access to past medical records. The study population is more representative of urban children, and the rural population could not be included due to practical difficulties.

## Conclusions

Obesity, particularly central obesity, has consistently been linked to hypertension and an elevated risk of cardiovascular problems. In this study, there is a significant association between sex and hypertension, with a male preponderance. Furthermore, the study also revealed a significant correlation between obesity and hypertension. It was also concluded specifically that adolescents with central obesity are more likely to have hypertension. Adolescents who are prone to obesity need early interventions and lifestyle modifications to reduce the chances of developing hypertension at a younger age, which can lead to life-threatening cardiovascular complications and sudden death. In the post-coronavirus disease era of 2019, there has been a disproportionate explosion in the prevalence of obesity in children in general. Specific, goal-oriented measures need to be implemented in schools to halt the increase in obesity prevalence and hence the serious side effects like hypertension.
